# The history of Chagas disease: reflections on science in action

**DOI:** 10.1590/0074-02760200372

**Published:** 2022-05-16

**Authors:** Simone Petraglia Kropf, Nísia Trindade Lima

**Affiliations:** 1 Fundação Oswaldo Cruz-Fiocruz, Casa de Oswaldo Cruz, Departamento de Pesquisa em História das Ciências e da Saúde, Rio de Janeiro, RJ, Brasil

**Keywords:** Chagas disease, history of science, social studies of science

## Abstract

Approaching from the perspective of the history and social studies of science, the article analyses some aspects of the early history of Chagas disease, from its discovery through initial research. It is our goal to show that historians of science can explore this topic as a way not only of remembering and narrating past events but also of examining the processes through which science is produced. To this end, we present five basic precepts that have guided historical and sociological studies of “science in action”: science as a collective endeavor, as a social activity, as a set of practices, as a process that involves controversies, and as a formative process. By examining the topic in the light of these five points, we demonstrate how the history of this successful research tradition can lead us to broader reflections about the complex dynamics interweaving science and society.

The 1909 discovery of American trypanosomiasis by physician and researcher Carlos Chagas, of the Oswaldo Cruz Institute (Instituto Oswaldo Cruz/IOC), was a milestone in the history and memory of Brazilian science and has become an internationally recognised emblem of a successful research tradition. In 2020, as the Oswaldo Cruz Foundation (Fundação Oswaldo Cruz/Fiocruz) celebrates its 120th anniversary, this scientific accomplishment has earned new acknowledgement from the global community, with the World Health Organization (WHO) inaugurating World Chagas Disease Day on April 14, the date on which Carlos Chagas identified the first human case of a *Trypanosoma cruzi* infection in the small town of Lassance, Minas Gerais. In establishing this commemorative date, it is WHO’s stated goal to encourage awareness about the people affected by this and other neglected diseases in Brazil and elsewhere around the world.

On occasions like this, historians are often invited to pronounce themselves and share their knowledge with physicians and scientists, people who were and still are active subjects of the historical process that is being remembered and celebrated, people for whom these memory rites are an important element of their identities. History is generally expected to offer lessons. Yet the words we offer here today, written for a journal that from its earliest days has figured centrally in the history of Chagas disease, are meant not as a teaching exercise but as an incentive for reflection. We would like to pose the following question: How do historians of science examine this topic as a way not just of remembering and narrating past events but also of embarking on a broader analysis of the processes through which science itself is produced? Before moving on to five topics that suggest some pathways for answering this question, we would like to explain in general lines our guiding theoretical and methodological perspective.

As historians, in the academic and professional sense of the term, we do not adhere to the more traditional view that long deemed science the product of individual “geniuses” who gradually unveil phenomena of nature based purely on method and experimentation. Since the closing decades of the twentieth century, the history and social studies of science have held that science is an activity produced as part of specific, concrete social and cultural dynamics, which apply not only to “external” conditions but also to the production and validation of ideas and theories.^1^ ^,^ ^2^ ^,^ ^3^ ^,^ ^4^ ^,^ ^5^ ^,^ ^6^ ^,^ ^7^ ^,^ ^8^

In today’s current climate of denialism, it is worth clarifying that when we say science is a social activity, it in no way means we are casting doubt on its status and credibility as an activity that produces authoritative knowledge consensually accepted as truthful.^7^ What it does mean is that we recognise science as a complex process entailing “reciprocal agency”^4^ between nature and society, in which the objective dimension of *facts* about nature does not invalidate the active role of the knowing subject and the meaning of acts of knowledge as a dimension of culture. It is precisely because we perceive science to be a human, social enterprise that we can identify and reflect on the mechanisms that endow it with strength and credibility, along with the mechanisms that seek to undermine it.^7^

That said, let us move on to our first point.

**Science as a collective enterprise**

Individual action is inarguably a vital dimension of history and social life. However, historians and social scientists have come to insist that science, like other forms of knowledge production, is essentially a collective activity, one not realised by isolated individuals but by concrete communities and groups that share ideas, practices, procedures, convictions, values, and spaces.^1^ ^,^ ^2^ ^,^ ^3^ ^,^ ^4^ ^,^ ^5^ ^,^ ^6^ ^,^ ^7^ ^,^ ^8^ As posited by Ludwik Fleck, whose theoretical proposition has become a classic reference in the history and social studies of science, the production of scientific facts always takes place within the realm of a “thought collective”, that is, “a community of persons mutually exchanging ideas or maintaining intellectual interaction” that provides the means for “the historical development of any field of thought, as well as for the given stock of knowledge and level of culture”.^8^

Turning to our topic of concern here, we can perceive the collective nature of science manifested in the very configuration of laboratories now conducting research on Chagas disease, where multidisciplinary teams of scientists work together while also maintaining ties to a transnational network of other laboratories. Yet when we look at the roots of this research tradition, we often notice Carlos Chagas portrayed as a lone individual who successfully achieved remarkable “feats” (a word that evokes the sense of heroism attached to his image), someone who acted practically on his own when he discovered and then conducted subsequent research into the disease bearing his name.

If we are to envision Carlos Chagas as part of a thought collective, we must do more than merely acknowledge the team of noteworthy collaborators who contributed to the great endeavor of investigating American trypanosomiasis on its various fronts, such as Ezequiel Dias, Arthur Neiva, Gaspar Vianna, Eurico Villela, César Guerreiro, Astrogildo Machado, and Carlos Bastos Magarinos Torres. The journey that took Chagas to the town of Lassance and to his celebrated discovery was a product of his membership in a very specific group of scientists, then engaged in building the IOC as a research centre in microbiology and tropical medicine.^9^ ^,^ ^10^ ^,^ ^11^ ^,^ ^12^ ^,^ ^13^ These were still fledgling disciplines, even in Europe, and to be part of them meant sharing new ideas on the causes of diseases and their means of transmission, as well as new protocols, techniques, practices, and spaces for knowledge production. It also meant embracing new beliefs and values regarding the social importance of science.^14^ All of this had its own unique features within the historical and social context of the young Brazilian republic, which was stamped by a colonial, slavery heritage and agricultural export economy.^11^ ^,^ ^15^

When Chagas finalised his medical studies in Rio de Janeiro by submitting a graduation thesis on haematological aspects of malaria in 1903 - the same year Oswaldo Cruz was named head of Brazil’s federal public health services - he wrote that he felt confident that the resources of “laboratory medicine” furnished a new paradigm for addressing the country’s health problems.^16^ Under the advisorship of Oswaldo Cruz, Chagas had conducted his thesis research at the recently opened Federal Serum Therapy Institute (Instituto Soroterápico Federal, renamed the Oswaldo Cruz Institute in 1908). This study inaugurated his career as a scientist within a collective that extended beyond the walls of the new institute, which from its birth had firm ties to scientific networks abroad (especially to German scientists), whose knowledge of African trypanosomiasis would prove fundamental to the discovery of Chagas disease and later research.^9^ ^,^ ^10^ ^,^ ^11^ ^,^ ^12^ The young researcher who investigated triatomines in Lassance because he suspected they might be the vector of a parasitic disease was able to acquire his repertoire of knowledge and practices precisely because he was a member of that collective. Once he had earned renown for his discovery of American trypanosomiasis and his related research and had become acknowledged as the prestigious heir to Oswaldo Cruz, Chagas was able to rally a network of collaborators and allies, not only in the laboratories where the disease was investigated but also in the social and political spaces that were stage to debates over the challenges facing the Brazilian nation and the directions it should follow.^11^ ^,^ ^13^ ^,^ ^17^ ^,^ ^18^

According to Bruno Latour,^19^ ^,^ ^20^ science is formed by a network of “heterogeneous associations” wherein scientists fulfill their parts alongside a wide array of other actors, human and non-human alike. What at first glance might appear to be a rather straightforward statement is actually an invitation for us to demystify the heroic notion of the “genius scientist”, not with the purpose of negating anyone’s merits or unique qualities but of impelling us to contemplate the complexity of the groups these scientists belong to and marshal. Who participates in these groups, and who does not? What do members have in common and what do they not? What are the dynamics through which these scientists gain or lose strength as they weave these networks? These essential questions can guide our understanding of “science in action”.^19^

**Science as a social activity**

The second point we wish to make derives from the first: science, as a collective activity, is built and acquires legitimacy within its own “internal” spaces (read: laboratories) but also within the “external” spaces of social life. Historians and social scientists have in fact questioned the very existence of any such boundaries, arguing that the social dimension of science lies both in the environment and in the content of science production, since it involves knowledge and practices shared and validated by concrete - and therefore socially configured - communities.^1^ ^,^ ^2^ ^,^ ^3^ ^,^ ^4^ ^,^ ^5^ ^,^ ^6^ ^,^ ^7^ ^,^ ^8^ ^,^ ^19^ ^,^ ^20^

In the case of Carlos Chagas’s research, the entwining of cognitive and social aspects is appreciable in the very way he produced and communicated the ideas through which he characterised the disease.^11^ ^,^ ^17^ ^,^ ^18^ From his earliest work, Chagas considered the disease to be medical-biological and social at once. Framed by the theoretical and methodological repertoires of tropical medicine and by the IOC’s institutional project,^9^ ^,^ ^10^ the new trypanosomiasis (whose African counterpart was a priority for European colonialism) was from the very beginning defined as a major public health issue and was increasingly cast as a concern critical to the expansion and interiorisation of republican modernisation in Brazil.^11^ The science then being institutionalised at the IOC thus discovered “a new morbid entity in man”,^21^ one that concomitantly revealed “another Brazil”, completely different from the “modern country” celebrated by the elites in the city of Rio de Janeiro, which had been reshaped to be the capital of the Belle Époque.^11^ ^,^ ^22^ In Brazil’s poor, rural *sertão* regions, American trypanosomiasis and other endemic diseases were the organic expression of poverty and abandonment stemming from the absence of public health services. Carlos Chagas defended this idea in virtually all his pronouncements and lectures. In 1910, he said: “Will it be possible, within public hygiene, to find efficacious ways of attenuating this affliction? We believe so, if this problem - most certainly a problem of the State and of humanity - becomes the concern of a scientifically well-guided statesman”.^21^

The disease itself was thus to frame a project for the society and the nation, one that found expression in Chagas’s engagement in the rural sanitation movement, which brought together scientists, intellectuals, and politicians to call for the Brazilian State to implement public policies that would serve the ailing people of the country’s interior.^22^ After Chagas was appointed head of federal public health services in 1919, two years after replacing Oswaldo Cruz at the helm of the IOC, he strove to enforce these guidelines through a substantial expansion of public health policies and services in the fight against rural endemic diseases.^23^

This was a key period in the construction of the IOC’s institutional identity, which intertwined academic excellence with social commitment to public health.^9^ ^,^ ^10^ ^,^ ^11^ We must bear this moment in mind as we ponder the other historical circumstances under which this identity was strengthened over the course of Fiocruz’s 120 years. Furthermore, in studying this moment we are summoned to grasp the more general understanding that science, whatever its format, always incorporates values, projects, and commitments as a part of the social world and is always a key element in defining this world.

**Science as a practice**

Our third point is that science, like other forms of knowledge, concerns not only the realm of ideas but also a set of concrete practices.^1^ ^,^ ^2^ ^,^ ^3^ ^,^ ^4^ ^,^ ^5^ ^,^ ^6^ ^,^ ^7^ ^,^ ^8^ In contrast with a long-enduring tradition of epistemologists who contended that scientific theories and facts should be analysed within the pure domain of methodological procedures by which they could be verified experimentally, since the 1960s and 1970s historians and social scientists have pointed out that, no matter how objective any given scientific fact or theory, we must not disregard the subjects who produced it and the concrete, specific circumstances that made it possible. The challenge has been to shift the focus of analysis from science’s “finished” products to the roads by which they arrived there - in other words, to shift focus from “ready” science to “science in action”,^19^ observing the practices as well as the places where this process transpires.

When we address the road taken by Chagas disease research from this perspective, we are prompted to explore such factors as the IOC expeditions, which the young institution soon began conducting to study and combat the health problems then jeopardising infrastructure works vital to republican modernisation.^15^ ^,^ ^22^ ^,^ ^24^ Historical narratives pertaining to the discovery of American trypanosomiasis often mention that Chagas’s famed visit to Lassance took place during his third malaria-fighting mission as a physician with the General Directorate of Public Health (Diretoria Geral de Saúde Pública). In 1905, the first of these missions had taken him to Itatinga, where malaria had halted work on the hydroelectric powerplant designed to furnish energy to the port of Santos, in the State of São Paulo. It was at this construction site that Chagas developed his theory of domiciliary malaria infection, the basis for his argument that transmission control efforts should concentrate on the use of insecticides inside homes, where they could combat the mosquito in its adult phase, instead of targeting larvae in standing water (Chagas eventually won international recognition for this contribution to the field of tropical medicine, although it was overshadowed by his research on American trypanosomiasis).^11^ ^,^ ^13^

Shortly thereafter, in 1907, the fight against malaria took Chagas to Xerém, in the Baixada Fluminense region of Rio de Janeiro State, where work was underway on the capital’s water supply system. On the third of these expeditions, Chagas traveled to northern Minas Gerais, between the towns of Corinto and Pirapora, where he led the fight against the malaria epidemic then assailing workers on the Estrada de Ferro Central do Brasil railroad, which, as Brazil’s main coffee shipping route, was being extended from Rio de Janeiro to northern Brazil. The ensuing events are quite well-known in the historical narratives about the discovery of Chagas disease: While Chagas was busy fighting malaria in the region, an engineer for the railroad warned him about the local triatomines. Knowing that blood-feeding insects in buildings can be vectors of parasitic diseases, the scientist examined the bugs and identified *T. cruzi*. Soon after, on April 14, 1909, he detected the first case of human infection.^11^ ^,^ ^12^ ^,^ ^13^ But historians of science do more than just recount these malaria campaigns as the “precursors” to Chagas’s discovery; they bring to light the specific social and cognitive environment that molded the field science practiced by Chagas and other researchers in the realm of tropical medicine.^11^ ^,^ ^22^ ^,^ ^24^

Field sciences were long seen as a preliminary stage of research, epistemologically secondary to the type of science practiced in the laboratory; that is, the field was considered a place expressly dedicated to collecting raw material for examination and transforming it into knowledge under controlled laboratory conditions.^25^ The *spatial turn* in historiography^26^ has problematised these boundaries, recognising the value of the links between these places as dimensions of “science on the move”,^25^ while likewise underscoring the specificities of the practices entailed in producing science in the field, where knowledge production occurs under multiform, heterogenous conditions within an environment displaying elements of both nature and culture. In the case of Chagas disease, the “hybrid space” of the field^25^ ^,^ ^26^ was not a waiting room outside the IOC’s laboratories. It was the concrete setting where these scientists were enabled to produce knowledge about the new illness and about the meaning frames that defined it as a nosological entity where elements of nature (vector, pathogen, its action in the human body) are inseparable from social elements (wattle-and-daub houses, their inhabitants, living conditions).

Materialised in the physical, social, and cultural universe of the *sertão*,^22^ these frames from the field played a decisive role in the production of knowledge about the illness in other spaces, that is, in the laboratories and hospital infirmaries where scientists identified the disease’s clinical and etiopathogenic features and also in the auditoriums and public platforms where scientists proclaimed the centrality of Chagas disease as a major public health concern for Brazil. Regarding this last point, we must remember that when Carlos Chagas assumed his public posts - as IOC director (1917-1934) and as head of federal public health services (1919-1926) - he was not exercising a separate side of himself. Rather, these positions (and the practices and places related to them) were deeply linked to his own identity and work as a scientist; furthermore, they had a direct impact on research on Chagas disease. These prominent offices also lent visibility to the scientist’s characterisation of American trypanosomiasis as an emblem of the “diseases of Brazil”^11^ and of tropical medicine, making his tenures there a critical ingredient in strengthening, and sometimes weakening, the scientific statements that defined Chagas disease as a medical and social problem, as we will see later on.

Over the course of the twentieth and twenty-first centuries, research on Chagas disease - like the scientists studying it - would inhabit many other spaces. At the same time, this research also came to comprehend new cognitive, social, and institutional practices and places, involving varied yet always complex connections between local, regional, national, and global dimensions.^11^ ^,^ ^18^ Reflecting on these processes and their specific historical features can valuably enhance our understanding of the pathways taken by this research tradition in the past and those it may take in the future. 

**Science as a winding road**

In these thoughts about science in action, we would like to make a fourth point, having to do with the idea that science does not follow a progressive, linear path towards the revelation of truth, but makes its way forward through clashes, controversies, and negotiations, as scientists pursue a stable foundation for consensus.^1^ ^,^ ^2^ ^,^ ^3^ ^,^ ^4^ ^,^ ^5^ ^,^ ^6^ ^,^ ^7^ ^,^ ^8^ ^,^ ^19^ This topic is particularly dear and meaningful to those who honor the memory and history of Chagas disease, given the controversy waged inside the National Academy of Medicine (Academia Nacional de Medicina) in 1922 and 1923.^11^ ^,^ ^27^ Carlos Chagas’s preeminence as a symbol of Brazilian science and medicine is so great that scholars and physicians who identify professionally with his heritage have called Chagas’s opponents in the fray “detractors”. In their eyes, the scientist’s opponents acted out of a sense of rivalry or pure and simple envy of the heir who stepped into Oswaldo Cruz’s shoes as director of the IOC and federal public health services. This perspective was defended by some of his collaborators at the time of the controversy and further disseminated later, when his youngest son, Carlos Chagas Filho, published a biography of his father.^28^ The book’s none-too-surprising personal slant coloured subsequent discussions, even spurring accusations against the “perpetrators” of the attack, led by Afrânio Peixoto, who held the chair in hygiene at the Rio de Janeiro School of Medicine.

As historians, we would like to call attention to the precept expressed in this episode: the production of science is always marked by controversies. In no way does this belittle Carlos Chagas; rather, it humanises him as a real, concrete actor within history. Furthermore, documental research - the historian’s essential task - affords insight into the significances of this controversy at the time it unfolded. This watershed moment in the history of Chagas disease has been analysed in depth elsewhere,^11^ ^,^ ^27^ so for the purposes of this article, we will limit ourselves to presenting our argument: factors both scientific and political (in various senses of the term) were deeply enmeshed during this controversy.

The scientific factors pertained to valid research questions concerning some of Chagas’s statements about the clinical presentation of the disease, especially about the correlation between endemic goiter and *T. cruzi* infection. These questions in turn raised doubts about the epidemiological expression of the disease. Since no stable methods were yet available for diagnosing the chronic phase of the illness, these uncertainties made it hard for Chagas and his collaborators to firm up a consensus. Regarding political aspects, while the disputes waged in arenas both inside and outside the IOC (cited by Carlos Chagas Filho)^28^ are certainly a factor worth considering, they are not the only one. Another decisive element - one that exemplifies the interweaving of the cognitive and social aspects of science - was the controversy between those who advocated and those who rebuffed tropical medicine as a specialty, in a process that reveals cognitive, institutional, and political demarcations within the field of Brazilian medicine.^11^ ^,^ ^27^ ^,^ ^29^ ^,^ ^30^ When Afrânio Peixoto classified American trypanosomiasis as a mere “illness from Lassance”, he was challenging both the position that Chagas disease had attained as an emblem of Brazilian biomedical research as well as the political meanings ascribed to it by the rural sanitation movement. In Peixoto’s view, the idea of a country beset by tropical illnesses fed into longstanding European biases about “the backwards tropics” and damaged the country’s image abroad, frightening off capital and immigrants.^11^ ^,^ ^27^ ^,^ ^29^ ^,^ ^30^

In short, this controversy was more complex than a movement fueled by personal, individual rivalry alone, instead encompassing disagreements between actors, practices, and places in the medical and social fields of the day. This is a fine example of how science in action is produced in spaces much noisier and through dynamics much more complicated than we might think if our gaze is limited to moments when agreement and consensus have already been firmly established.^19^ Clarifying these disputes and their motivations and significances is important, but not with the sole purpose of allowing those who value knowledge of the past to peer at them from another angle. Here, history must encourage everyone, scientists and non-scientists, to think in the present about the places and positions they occupy within the complex web that binds science and society together. Understanding the winding roads that lead to consensus in science strengthens our capacity to explain why we must trust it.^7^

**Science as a formative activity**

Approaching from this same perspective, let us present our final point in this endeavor to make the history of Chagas disease an invitation for biomedical scientists, in dialogue with historians and social scientists, to examine processes of knowledge production with fresh eyes. We are talking about the centrality of teaching and training in lending continuity to science and also in transforming it. Once again, let us point out that this is not a “natural” process but one defined by specific circumstances in distinct historical contexts.

In 1925, when Carlos Chagas assumed the newly established chair in tropical medicine at the University of Rio de Janeiro’s School of Medicine, the medical field had yet to reach a consensus about the teaching of this discipline, as mentioned earlier. Moreover, despite continued research into Chagas disease, there remained uncertainties that had grown out of the controversy within the National Academy of Medicine. It was in his capacity as a professor that Chagas doggedly asserted the value of studying tropical diseases, including trypanosomiasis. In the Brazilian context, this meant emphasising the importance of rural endemic diseases, personified in the figure of Jeca Tatu, a fictional character who had been rendered non-productive by tapeworms and who thus could be redeemed by medicine. Jeca was created by writer Monteiro Lobato, he himself an advocate of the rural sanitation movement.^22^ ^,^ ^23^

In his classes, as in his lectures, Chagas avowed that physicians should be trained in the diagnosis and treatment not only of the diseases presented by patients in their urban offices but also of what he labeled the “diseases of Brazil”.^11^ ^,^ ^30^ He considered these illnesses the major public health issue for Brazilians, “avoidable diseases”, as they were called. Further according to the scientist, meeting this challenge would require the conjoining of clinical knowledge and practice with laboratory knowledge and practice. Just as he had trained under the paradigm of experimental medicine, influenced by such masters as Francisco Fajardo, Miguel Couto, and Oswaldo Cruz, Carlos Chagas - already a master in his own right - sought to recruit new generations to carry on the national project taken up by IOC science. While Chagas himself had encountered various forms of resistance within his own alma mater, the chair in tropical medicine would become a central space for the reproduction of this nascent research tradition, further ensuring its institutionalisation elsewhere.^11^ ^,^ ^30^

The concept of an imperative bond between laboratory and clinical practice was to provide the main pathway to settling the uncertainties and filling in the gaps made apparent during the Academy of Medicine controversy; this was also how the disease came to be recognised as a public health issue and earn a place on the Brazilian State’s political agenda in the 1940s and 1950s.^11^ ^,^ ^18^ This process of recognition got underway when expertise gleaned from tropical medicine and parasitology was combined with that from clinical practice, in particular cardiology following the 1943 creation of the Centre for the Study and Prevention of Chagas Disease (Centro de Estudos e Profilaxia da Moléstia de Chagas), an IOC research post in the town of Bambuí, Minas Gerais. The post’s director, IOC researcher Emmanuel Dias, was a leading disciple of Carlos Chagas. When Dias learned that the cardiologist Francisco Laranja had vast experience as a physician with the Institute of Pensions and Retirement for Industrial Workers (Instituto de Aposentadoria e Pensões dos Industriários), the former invited the latter to join his research centre team. Thanks to state-of-the-art electrocardiography techniques not available during Chagas’s time, this laboratory-clinic partnership enabled the recognition of chronic Chagas cardiomyopathy as a prime clinical manifestation of Chagas disease (an idea defended by Chagas). This forged a new consensus regarding Chagas’s ideas, in turn allowing the topic to draw the attention of other physicians and consequently enjoy new social visibility.^11^ ^,^ ^18^

It was against the backdrop of Getúlio Vargas’s project to build a “new nation”, anchored in the figure of the “new Brazilian worker”, that Chagas disease inspired renewed concern as a medical and social issue and as a malady prejudicial to the health and productivity of rural workers. In 1950, with the introduction of residual insecticides and under the technical guidance of Dias, the Ministry of Education and Health (Ministério da Educação e Saúde) launched Brazil’s first vector control campaign, in the region known as the Minas Gerais Triangle. From then on, the question of Chagas disease was taken up by various research groups at other medical schools in Brazil. The generation following that of Emmanuel Dias thus had many disciples, who carried on the tradition while also innovating it. Among this new generation was Dias’s own son, João Carlos Pinto Dias, a key protagonist in efforts to track and control the disease.^11^ ^,^ ^18^

**In conclusion**

The topics we have addressed here demonstrate how the discovery of Chagas disease was more than a milestone in the history of Brazilian science. This event and its ensuing developments afford us a rich opportunity to examine scientific work itself, as historians producing knowledge that transcends an inventory of the past undertaken upon the occasion of commemorations or memory rituals. The knowledge produced by historians, through the field’s specific theoretical references and research protocols, thus plays a valuable social role, stimulating scientists from different fields to reflect on their roles, identities, practices, projects, and place in society - in the past but also in the present.

In 2020, the year that Fiocruz celebrates its 120th anniversary and WHO launches April 14 as World Chagas Disease Day, it is clearer than ever before that history has a worthwhile contribution to make. A good way to commemorate the research tradition that earned Brazilian science international prominence is to highlight how it has from its very inception maintained tight bonds with a bigger social project: to raise public awareness and seek solutions for the serious public health problem that struck Berenice - the little girl in whose blood Chagas identified *T. cruzi* on April 14, 1909 - and that continues to plague millions of people around the world today. The current Covid-19 pandemic, as a public health emergency of devastating proportions, situates us in a moment of grave scope and implications. Let us look to the past with our feet rooted firmly in the present, retrieving the history of Chagas disease as an example of the interrelationships between science, health, and society, not simply examining how this history unfolded over time but asking ourselves how it might shed light on the paths we now want to chart.

## References

[1] Pickering A (1992). Science as practice and culture.

[2] Golinski J (1998). Making natural knowledge: constructivism and the history of science.

[3] Gavroglu K (2007). O passado das ciências como história.

[4] Maia CA (2015). História, ciência e linguagem. O dilema relativismo-realismo. Mauad X.

[5] Condé MLL (2017). Um papel para a história. O problema da historicidade da ciência.. UFPR.

[6] Lightman B (2016). A companion to the history of science. John Wiley & Sons Incorporated.

[7] Oreskes N (2019). Why trust science?.

[8] Fleck L (1979). Genesis and development of a scientific fact. University of Chicago Press.

[9] Stepan NL (1976). Beginnings of Brazilian science: Oswaldo Cruz, medical research and policy, 1890-1920.

[10] Benchimol JL (1990). Manguinhos do sonho à vida: a ciência na Belle Époque.

[11] Kropf SP (2009). Doença de Chagas, doença do Brasil: ciência, saúde e nação (1909-1962).

[12] Kropf SP, Sá MR (2009). The discovery of Trypanosoma cruzi and Chagas disease (1908-1909): tropical medicine in Brazil. Hist Cienc Saude-Manguinhos.

[13] Kropf SP, Lacerda AL (2009). Carlos Chagas, a scientist of Brazil.

[14] Arnold D (1996). Warm climates and Western medicine: the emergence of tropical medicine, 1500-1900.

[15] Benchimol JL, Silva AFC (2008). Ferrovias, doenças e medicina tropical no Brasil da Primeira República. Hist Cienc Saude-Manguinhos.

[16] Chagas C (1903). Estudos hematológicos no impaludismo.

[17] Kropf SP (2011). Carlos Chagas science, health and national debate in Brazil. Lancet.

[18] Kropf SP (2013). Chagas disease in Brazil: historical aspects. In FR Gadelha, EF Peloso, editors. Chagas disease: still a threat to our world?. Nova Science Publishers.

[19] Latour B (1987). Science in action. How to follow scientists and engineers through society. Harvard University Press.

[20] Latour B (2005). Reassembling the social: an introduction to Actor-Network-Theory.

[21] Chagas C (1910). Nova entidade mórbida do homem. Brazil-Medico.

[22] Lima NT (2013). Um sertão chamado Brasil: intelectuais e representação geográfica da identidade nacional.

[23] Hochman G (2016). The sanitation of Brazil: Nation, State, and public health, 1889-1930.

[24] Lima NT (1998). Missões civilizatórias da República e interpretação do Brasil. Hist Cienc Saude-Manguinhos.

[25] Kohler RE, Vetter J (2016). The field. In B Lightman, editor. A companion to the history of science.. John Wiley & Sons Incorporated.

[26] Livingstone DN (2003). Putting science in its place: geographies of scientific knowledge. Chicago/.

[27] Kropf SP (2009). Carlos Chagas e os debates e controvérsias sobre a doença do Brasil (1909-1923). Hist Cienc Saude-Manguinhos.

[28] Chagas C (1993). Meu pai.

[29] Edler F (2010). Medicina tropical: uma ciência entre a nação e o império. In A Heizer, AAP Videira, editors. Ciência, civilização e República nos trópicos.

[30] Kropf SP (2015). Carlos Chagas e as doenças do Brasil. In G Hochman, NT Lima, editors. Médicos intérpretes do Brasil.. Hucitec.

